# The Influence of Buffer Layer Type on the Electrical Properties of Metallic Layers Deposited on Composite Textile Substrates in the PVD Process

**DOI:** 10.3390/ma16134856

**Published:** 2023-07-06

**Authors:** Marcin Lebioda, Ewa Korzeniewska

**Affiliations:** Institute of Electrical Engineering Systems, Lodz University of Technology, Stefanowskiego 18, 90-537 Lodz, Poland; marcin.lebioda@p.lodz.pl

**Keywords:** thin films, thin layers, textronics, wearable electronics, textile substrates, PVD

## Abstract

In the era of developing wearable electronics, the miniaturization of electronic systems and their implementation in the textile industry is one of the key issues. For this reason, it is important to select the appropriate textile substrates upon which it is possible to produce electroconductive structures, as well as their selection from the point of view of the electrical parameters’ stability. For this purpose, research related to the effect of heating a substrate on the resistance of the structures produced in the process of physical vacuum planting was conducted. Textile composites with a buffer layer made of polyurethane, Teflon, and acrylic were used as substrates in the tests. Such layers are an integral part of textile composites and a necessary element for producing structures with continuous electrical conductivity. The conducted tests showed that a buffer layer made of polyurethane (thermal conductivity, e.g., PERMACOL 5450 resin 0.16 W/mK) heated to 15 °C above room temperature was a layer that introduced changes into the surface resistance of the structures. The resistance values of the samples produced on a substrate containing a buffer layer of polyurethane varied in the range of 9–23%, depending on the manufacturer of the composite in the case of a self-heating mode, and in the case of an external heating mode, these changes were smaller and ranged from 8 to 16%. Such a phenomenon occurred regardless of the type of applied metal, and this was not observed in the case of composites with a Teflon or acrylic sublayer. For this reason, it is necessary to take into account the fact that textronic structures made on substrates containing a polyurethane layer may change the surface resistance depending on the temperature. The electrical parameters of such structures were checked by heating the structure using an external heater and self-heating mechanism. The same phenomenon was observed in both cases.

## 1. Introduction

The development of electronic technology is conducive to the continuous search for solutions regarding the miniaturization of systems, as well as for materials upon which such systems can be created. Flexible materials, with a particular emphasis on textiles, are among the substrates of great interest. The combination of a textile substrate with elements of wearable electronics is widely used in various fields of life, such as medicine, tourism, sport, or the military industry [1,2,3,4,5]. This issue is a complex one, requiring the appropriate selection of both the substrate and the technique of applying thin electroconductive layers. Thin films play the role of passive elements, as do the connections between the elements of the systems.

There are some ways of producing thin metallic layers on substrates. They include: inkjet printing, a method of producing conductive layers using ink containing metal particles with a low resistivity, usually silver [6,7]; sputtering, which uses plasma to deposit thin metallic layers on flexible substrates [8,9,10,11]; chemical vapor deposition (CVD), where thin metallic layers are deposited onto a flexible substrate with gaseous chemicals reacting with each other on the surface of the substrates, where they form a metallic deposit [12]; an electrochemical process known as electrodeposition, in which metallic ions are deposited onto a substrate [13,14]; spray-coating, a technique in which thin metallic layers are deposited onto a substrate with a sprayer that sprays a solvent containing metallic particles [15]; and physical vacuum deposition (PVD), in which thin layers are deposited onto the surface of a material using the vacuum deposition technique [16]. In PVD technology, the process begins with placing the base material upon which the layer is deposited. A source of evaporating material is placed in the chamber and heated to a temperature sufficient for boiling. The process takes place in a low-pressure chamber to avoid contamination and provides a controlled environment. During the deposition process, the metal vapors, as a result of the boiling, detach from the source, having the appropriate energy to move, and are then deposited onto the substrate with a lower temperature and adhere to the surface of the target material, creating a nanometer metallic layer [9]. During the use of the created layers, as well as at the stage of their formation, microcracks and defects may appear that have a significant impact on the resistance of the produced structures [17].

These particles settle on the surface of the material and form a thin layer, which, depending on the porosity of the substrate, can be a layer with continuous electrical conductivity. An assessment of the smoothness of the substrate can be carried out, using, for example, the method of optical coherence tomography [18], which, compared to ultrasonic tomography [19] or electrical tomography [20], has a much higher resolution.

During the process, it is important to control the technical parameters, such as the temperature and pressure, which affect the quality and properties of the final layer.

Physical PVD deposition is a popular technique used in the electronics, optical, semiconductor, and medical industries to produce thin films with a high quality and accuracy. An improvement in the electrical properties of the produced structures can be obtained by modifying the surface upon which they are created with a laser beam [21]

One of the key problems related to the production of such structures is their adhesion to the substrate [22,23]. The impact of the thermal stability of the substrate and buffer layer on the quality of the thin layers is highlighted by, among others, a group dealing with thin films for wearable energy harvesting [24]. Prasad’s group paid attention to the thin-film adhesion and their degradation with temperature [25].

So far, the buckling and delamination of such thin films has been treated as a negative phenomenon that should be eliminated. However, literature reports have indicated the usage of the mentioned phenomenon in the development of flexible electronic devices [26,27,28,29].

For this reason, it is important to understand the phenomena occurring at the interface between the substrate and the created metallic layers. The quality of the interface is very important for ensuring reliable microelectronic devices [30]. Applying thin metallic structures in wearable electronics systems is also influenced by the connections made between the elements of the systems [31], so, due to previous work, the glued connections are used in current research.

The authors of this paper present the results of experimental research works related to the analysis of the changes in the resistance of electroconductive layers produced on a flexible textile composite substrate due to the Joule’s heat generated during the use of the layers.

## 2. Materials and Methods

### 2.1. Sample Preparation

Textile composites were used as the substrates for the creation of the metal electroconductive layers.

The first is a CORDURA^®^ (Invista, Kansas, MO, USA) material with a surface density of 195 g/m^2^—sample A (Table 1). It is a material with a high mechanical strength. Nylon threads are the basis for the production of this material. The threads are covered with a layer of polyurethane, which smooths the three-dimensional surface, enabling the creation of a continuous electroconductive structure. On a woven structure, without an additional buffer layer leveling the surface, it is not possible to produce an electroconductive structure in PVD technology. In addition, polyurethane fills the spaces between the nylon fibers and limits the possibility of their movement during the use of the material. The second selected material is a white modified Goretex membrane (130 g/m^2^). The bottom side of this material is extended with a layer of loosely braided fibers, which is an additional thermal barrier. Nanofibers are attached to the outer side of the membrane, covered with a thin Teflon foil. The use of such a membrane in clothing, while maintaining all the advantages of Goretex, improves the thermal comfort of its users. In the further content of the work, this material is marked as sample B—Membrane (Table 1).

The next tested substrate with a different buffer layer is a spun-bonded, non-woven polypropylene substrate with a surface density of 120 g/m^2^ and thickness of 0.52 mm, which is covered with an acryl layer—sample C (Table 1). 

In order to assess the effect of the intermediate layer on the electric properties of the thin metallic films created on the textile substrates, the authors of the paper prepared their own composite in the form of bamboo fabric soaked in polyurethane resin Permacol 5450. That kind of substrate is marked as sample D (Table 1). It should be noted that depositing a conductive metallic layer directly onto the bamboo material is not possible. The obtained layers are were due to the weave and structure of the material. That material was used only for the creation of the matrix for resin.

The next material is a Cordura equivalent called RAINCRUCIAL^®^ (Concordia, Waregem, Belgium). This material consists of nylon threads coated with polyurethane. The surface density of this sample is 155 g/m^2^—sample E (Table 1). Additionally, it has a Teflon finish and water-, dirt-, and oil-resistant fluorocarbon impregnation from Concordia.

Such a selection of substrates allows for a verification of the influence of temperature on the electrical properties of the textronic structures, depending on the intermediate layer between the three-dimensional textile structure and thin electroconductive layer.

**Figure 1:**
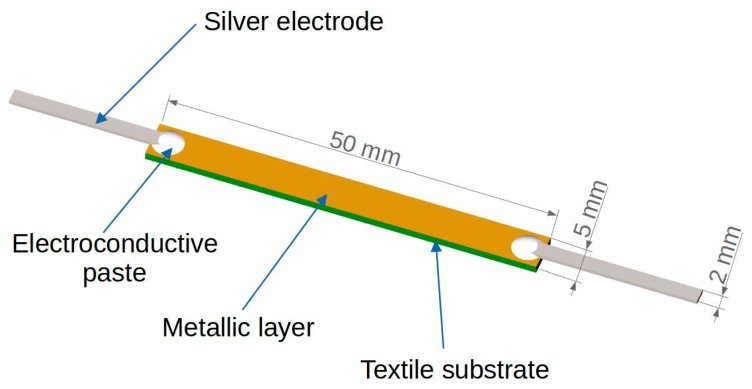
Schematic view of the sample geometry.

**Figure 2:**
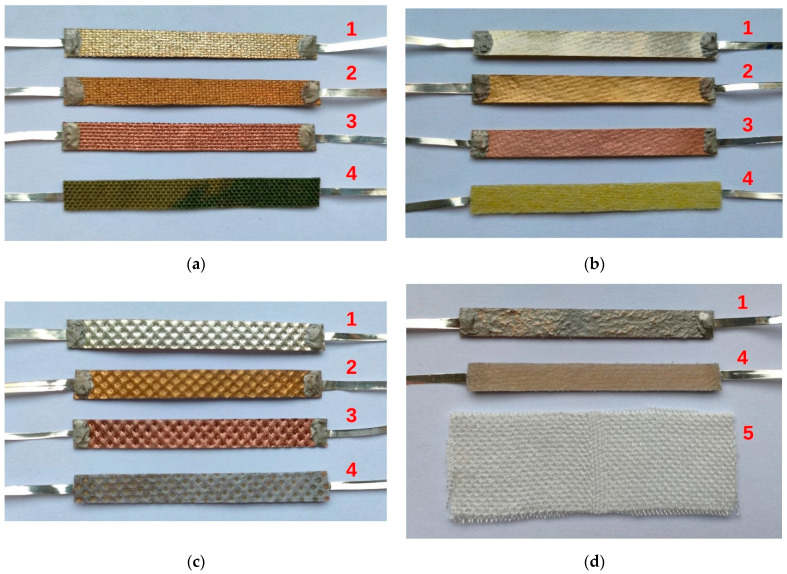

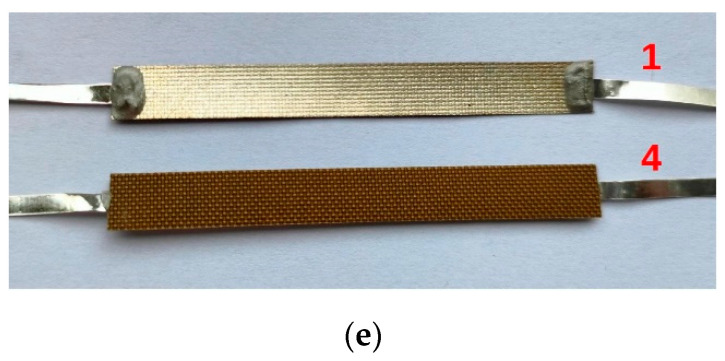
Views of samples (**a**) Cordura—sample A, (**b**) membrane—sample B, (**c**) non-woven—sample C, (**d**) composite—sample D, and (**e**) Cordura with PTFE—sample E, where 1—sample with Ag layer deposited, 2—sample with Au layer deposited, 3—sample with Cu layer deposited, 4—back side of the sample, and 5—bamboo fabric.

The substrates were conditioned at 20 °C for 24 h before the thin layer deposition process. Each substrate was cleaned with isopropyl alcohol and then placed inside a Classic 250 Pfeiffer Vacuum chamber (Wetzlar, Germany) at a distance of 6 cm from the tungsten boat in which the vapor sources were placed. Silver, gold, and copper with a purity of 99.99%, provided by Mennica-Metale Ltd. (Radzymin, Poland), were used as the deposited materials. The deposition process was started with an initial vacuum of 0.05 Pa (5.0 × 10^−4^ mbar) and lasted for 5 min for each sample. The placed substrates were rotated during the metal deposition process to ensure a uniform deposition of the metal vapors and thus the homogeneity of the thin layer. Current electrodes were glued to the prepared layers using ELPOX AX 15S conductive paste (Amepox Ltd., Lodz, Poland). This type of glue consists of two components based on epoxy resin. After mixing, the glue becomes a smooth paste of a bright silver color. The technical data of the glue are available on the producer’s website [32]. A joint with dimensions of about 3 × 5 mm was made between the thin metallic (Ag/Au/Cu) layer deposited onto the substrate and 35 μm metal foil (Figure 1 and Figure 2). 

Due to the flexibility of the substrates, the thickness of the produced layer was measured indirectly. The reference glass plate was also placed in one process alongside the target textile substrates. The thickness of the formed layer was measured using a Dektak 3ST profilometer (Veeco Plainview, New York, NY, USA). The analysis of the measurements of the layer thickness showed that its thickness was 250 nm ± 20 nm.

### 2.2. Instrumentation and Measurement Procedure

**Figure 3:**
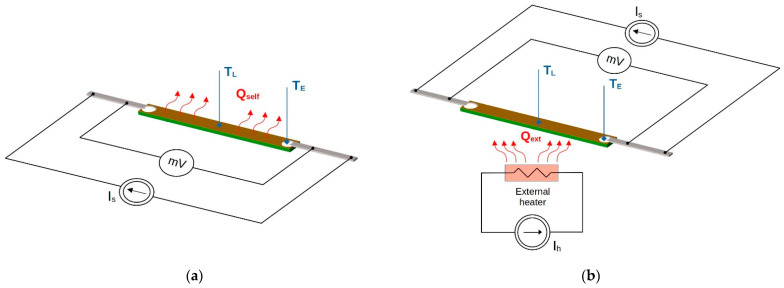
Measurement methods used in two modes of heat transfer (**a**) self-heating mode, and (**b**) external-heating mode.

The main research of the work was focused on an analysis of the thermal and electrical properties of the metallic layer deposited onto the textile substrate. Samples with the same geometry (Figure 1) were studied at room temperature under natural convection conditions. The experimental study was conducted in two modes of heat exchange, determined by the environmental conditions and type of heat source:

- Self-heating mode—the investigated sample was heated using Joule heat *Q_self_* generated by the electrical current in the sample (Figure 3a). In this case, the metallic layer deposited onto the textile substrate was the only heat source supplied from the controlled current source *I_s_*.
- External-heating mode—the investigated sample was heated using heat *Q_ext_* generated by the external heater placed under the sample (Figure 3b). A power resistor (15 Ω) supplied from the controlled current source *I_h_* was used as the heater. The sample was supplied from the controlled current source *I_s_*. The current in the sample was selected so that the temperature change associated with the current transport in the metallic layer was negligibly small. In addition, this configuration allowed the sample to be tested in mixed mode with two heat sources: self-heating and external.

The center of the samples was heated to ~15 °C above room temperature (*T_init_* = 20 °C) to bring the test conditions as close as possible to the typical usable temperature range for fabrics. This assumption allowed for a comparison of the samples, regardless of their resistance and type of metallic layer. To limit the influence of external factors, such as mechanical stress or uncontrolled heat transfer, the test samples were mounted in a special rack used in the previous authors’ work [31]. A special stand ensured that the sample was supported with thin threads without tension and with minimal thermal contact [31]. The sample and external heater were supplied from dual, independently controlled current sources HM8142 (Hameg Instruments, A Rohde & Schwarz Company, Frankfurt, Germany). An HP34420A Nano-Voltmeter (Keysight Technologies, Santa Rosa, CA, USA) was used to measure the sample voltage drop (Figure 3). The surface temperature of the sample was measured with two thin (*ϕ* = 0.12 mm) T-type thermocouples placed at the center (Figure 3, T_L_) and electrode/joint (Figure 3, T_E_) of the sample, respectively. Such an arrangement of the thermocouples allowed for an identification of the dominant heat source in the sample. Thin thermocouples are not a significant additional heat load and did not affect the thermal conditions. 

## 3. Results and Discussion

**Figure 4:**
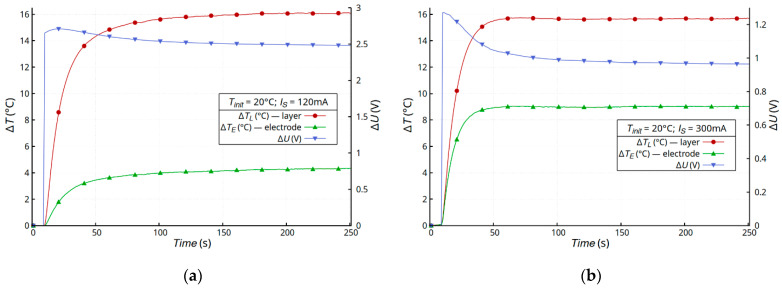

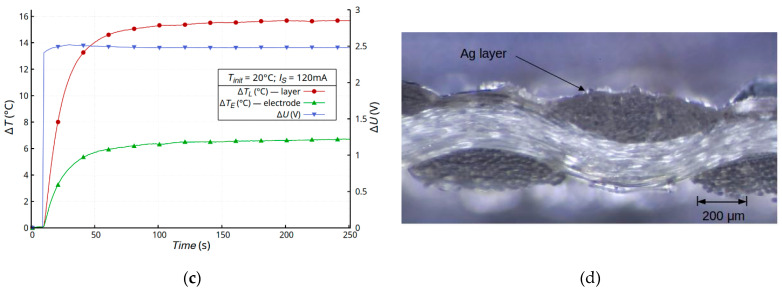
Time dependence of temperature changes Δ*T* and voltage drop Δ*U* on Cordura samples with a metallic layer of gold (**a**), silver (**b**), and copper (**c**), and a microscope image of the cross-section of a Cordura sample (**d**).

In electrical systems, any current flow through structures with non-zero resistance generates heat. For this reason, the authors investigated the effect of heat on the electrical properties of a created thin metallic layer deposited on a textile substrate with a room temperature resistivity of the order of a single Ohms/square. The samples were heated in a self-heating or external-heating mode under natural convection conditions. In the self-heating mode, the samples were heated for 4 min. During this time, the temperatures of the layer and joint were stabilized. Figure 4 shows the time dependence of the temperature changes and drop voltage on the Cordura substrates (sample A). The dynamics of the sample heating process were independent of the type of metallic layer and the temperature was constant after about 1 min. The layer was the dominant source of heat in all the samples (Δ*T_L_*), and the temperature changes in the joint between the electrode and layer (Δ*T_E_*) were at least two times lower. The initial increase in the voltage on the sample (Δ*U*) resulted from the increase in the temperature of the metallic layer. This is a typical dependence of metal resistance on temperature due to phonon scattering, when an increase in temperature causes an increase in metal resistance. After reaching constant values for the temperatures of the layer and electrode, atypical changes in the voltage on the sample were observed at a constant sample current. Despite the constant temperature of the sample, the voltage on the sample (Δ*U*) decreased.

Atypical changes in the layer resistance were observed for all metal types of the layers, with the strongest changes being ~25% for the silver layer (Figure 4b). It should be noted that each sample had a different resistance depending on the layer metal, but the generated power in each of the samples was the same, at about 0.3 W. We assumed that the observed effect should be associated with the thermal and mechanical changes occurring in the textile substrate and the integration of the metallic layer with the substrate. As was described in Section 2.1, Cordura is a composite substrate with a complex structure. The weave of nylon threads is covered with a thin layer of polyurethane [31]. In the case of Cordura, polyurethane plays a key role as a binder. The weave of nylon fibers is a structural element of the composite that determines the strength of the material. In this case, temperature changes had the strongest impact on the flexible polyurethane element, which can undergo thermal deformation. The process was inertial due to the heat transfer in the composite. The nylon matrix was less susceptible to temperature changes. To verify this hypothesis, two composites with different buffer layers were tested: membrane (sample B) and non-woven (sample C).

**Figure 5:**
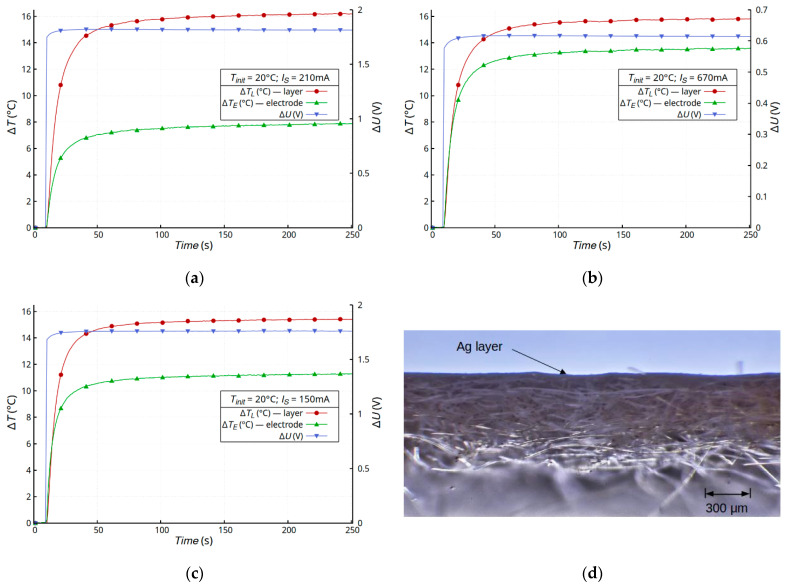
Time dependence of temperature changes Δ*T* and voltage drop Δ*U* on membrane samples with a metallic layer of gold (**a**), silver (**b**), and copper (**c**), and a microscope image of the cross-section of a membrane sample (**d**).

Figure 5 shows the time dependence of the temperature changes in the samples (Δ*T_L_*), electrodes (Δ*T_E_*), and drop voltage (Δ*U*) on the membrane samples (sample B). This composite consisted of a layer of compressed fibers bonded to a layer of Teflon (Figure 5d). The metallic layer was deposited directly onto the Teflon layer. Regardless of the metal layer, no unusual voltage changes were observed. The initial increase in the voltage on the sample (Δ*U*) was due to the increase in the temperature of the metallic layer, but, at a constant temperature, the voltage was also constant (Figure 5a–c). 

**Figure 6:**
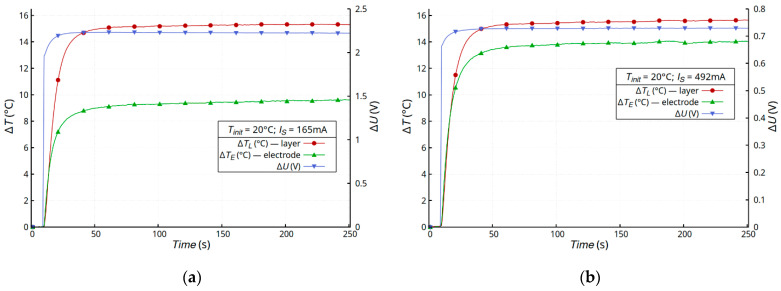

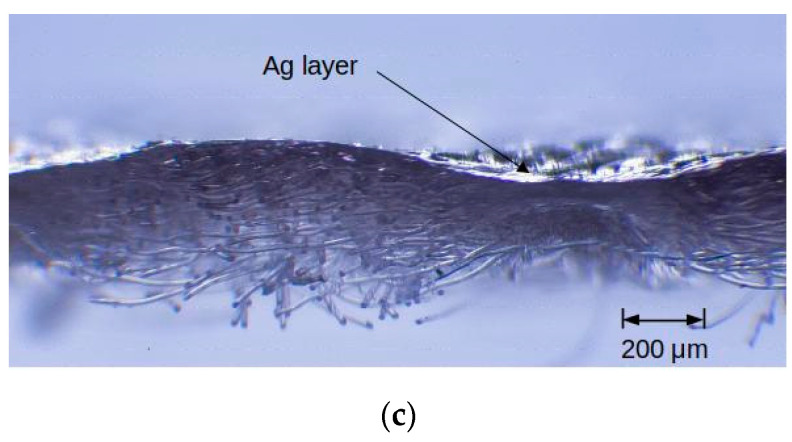
Time dependence of temperature changes Δ*T* and voltage drop Δ*U* on non-woven samples (sample C) with a metallic layer of gold (**a**), silver (**b**), and a microscope image of the cross-section (**c**).

Small and not very dynamic changes in the voltage should be identified with the transport of heat from the metallic layer to the highly porous fibrous substrate. Very similar results were obtained for the non-woven samples coated with an acrylic layer—sample C (Figure 6). This was due, among other reasons, to the similar structure of the composite (Figure 6c). A constant voltage at a constant sample temperature was observed. 

**Figure 7:**
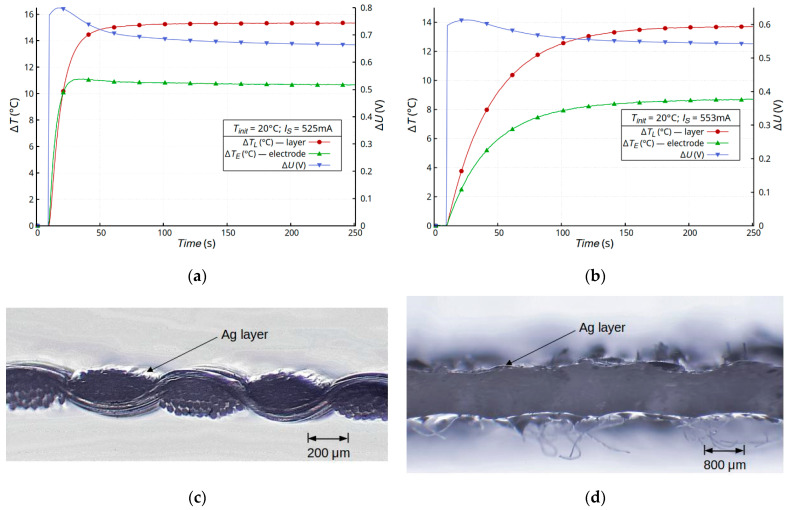
Time dependence of temperature changes Δ*T* and voltage drop Δ*U* on sample E (**a**) and sample D (**b**) samples with a silver layer and microscope images of the cross-section of a sample E (**c**) and samples D (**d**).

In order to confirm the role of the polyurethane layer in the process of changing the resistance of the deposited metallic layer, additional tests were carried out on two materials: a thin Cordura, sample E (Figure 7a,c), and a thick composite with a dominant polyurethane resin phase, sample D (Figure 7b,d). In both cases, atypical changes in the resistance of the metallic layer were observed, despite a significant difference in the volume of the materials. It should be noted that, in the case of the composite (Figure 7b), the thermal processes were much less dynamic, but a change in resistance (~12%) was observable. In addition, the effect of these changes in resistance was observed despite the significant differences in the surface profiles of the materials. The surface of the composite was very uneven and irregular, unlike the surface of sample E (Figure 7c,d). It should be noted that the metallic layer on substrate E was deposited directly onto the Teflon layer, which was the outer layer. Polyurethane was another layer in the composite structure, but it significantly affected the changes in the resistance of the sample (~17%) as a result of the temperature change.

The external heating of the samples was more uniform and caused less dynamic changes in the temperature and resistance of the samples. Placing the heater below the sample forced a laminar flow of the warm air stream and the indirect heating of the metallic layer. In this configuration, the metallic layer was substantially indirectly heated by the textile substrate. In order for the temperature change associated with the current transport in the metallic layer to be negligibly small, the current in the sample *I_s_* = 30 mA (the power generated in the sample was ~0.0045 W). Lower dynamics of thermal processes require a longer heating time in order to achieve a stable temperature. In the external-heating mode, the samples were heated for 20 min by the resistance heater powered by *I_h_* = 315 mA (Figure 3b).

**Figure 8:**
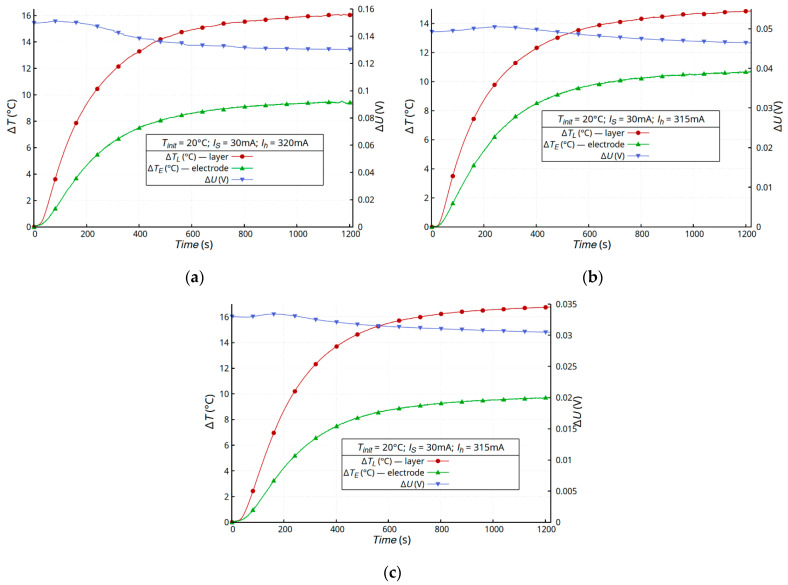
Time dependence of temperature changes Δ*T* and voltage drop Δ*U* on the sample A (**a**), sample E (**b**), and sample D (**c**) in the external-heating mode.

Figure 8 shows the time dependence of the temperature changes and drop voltage on samples A, D, and E. Samples with a silver layer, previously tested in the self-heating mode, were used for the study. The low dynamics of the heating process made it possible to limit the influence of the volume and heat capacity of the samples on the results. All the samples showed an atypical change in their layer resistance due to the temperature increase. After a slight initial voltage increase due to the increase in the metallic layer temperature, a slow voltage decrease was observed at a constant temperature (Figure 8).

The resistances achieved in the self-heating mode were lower. This difference was due to the dynamics of the heating process. It is difficult to compare the instantaneous values, because the test times were different due to the different dynamics of the heating process; for this reason, the values of the resistance in the steady state are given in Table 2. The values presented in Table 2 are for one typical sample of each kind.

In the self-heating mode, the layer was a surface heat source that heated the substrate. In the external-heating mode, the layer was heated indirectly by the substrate and convection in the air. 

The type of metal deposited onto the surface of the textile did not qualitatively affect the observed phenomena. Regardless of the used substrate, the silver layers had the best electrical properties. For all the samples with a silver layer, the difference between the temperature of the layer and electrode was the smallest. This was due to the relationship between the resistances of the layer and the joint. The silver layer had the lowest resistance and, to obtain a temperature increase of 15 °C, it required the highest current value *I_s_* > 300 mA; for this reason, a higher joint temperature was observed.

The observed resistance changes should not be overlooked, because in the extreme case, i.e., Cordura, they reached 25%. For all the tested samples containing polyurethane with an Ag layer, the changes exceeded 10%, regardless of the self-heating or external-heating test mode. It should be noted that the increase in the temperature should lead to an increase in the silver layer resistance of approx. 6%, which was observed in the initial phase of the heating process. The atypical decrease in resistance was a less dynamic process and resulted from thermo-mechanical changes in the substrate, specifically in the polyurethane layer.

The coordination between the polar groups of the polyurethane and metal could also affect the electrical properties of these complexes [33]. Metal coordination polyurethanes (MCP), which are formed in the case of zero-dimensional metal ions with a surrounding array of one-dimensional polyurethanes, exhibit interesting physical properties. Metal-polyurethane complexes, apart from their thermoplasticity, flexibility, and electrical conductivity, are also characterized by self-repair and memory, so that the polyurethane can be developed towards smart materials [34]. It should be noted that the specificity of the interaction between the metal ions and polyurethane can significantly expand the range of applications. The described mechanism could probably function in the samples produced by us, but it may occur when the metal layer is formed directly on the polyurethane. However, an atypical effect of changing the resistance was also observed for the samples where polyurethane was the intermediate layer, but metal was deposited directly onto Teflon (sample E). Therefore, based on the research on self-heating and external heating, we assume that the dominant thermomechanical processes in the substrate, specifically in the polyurethane layer, were responsible for the unusual changes in the resistance. 

The observed phenomenon indicates the need to take into account the influence of the type of composite buffer layer on the electrical properties of the textronic structures formed on their surface. Metallic thin-film structures change their resistance not only as a result of heating their structures, but also as a result of heating the substrate. During the designing of textronic structures, not only should the temperature coefficient of the metal resistance changes be taken into account, but so should the resistance changes resulting from the applied buffer layer in the composite used as a substrate. There is no description of the coefficient describing such changes in the literature and further work should be carried out to determine such a coefficient, not only using experiments, but also by developing numerical or functional values characteristic for a given material.

## 4. Conclusions

The production of wearable electronic structures on textile substrates requires not only the selection of an appropriate technique for applying conductive layers, but also an appropriate substrate that will be the carrier of the entire textronic system. For this reason, it is important not only to select a substrate with an appropriate surface structure, but also one with an appropriate mechanical strength. Among the many composite materials available on the market, Cordura, which was used in the presented research, has a good resistance to abrasion and tearing and is used in clothing with increased mechanical strength. A microporous membrane was also analyzed as a representative of composites made of foamed, porous PTFE, recognized as a waterproof, windproof, and breathable material for outdoor clothing and footwear. The composite material, which consisted of a non-woven fabric as a structural component and an acrylic top layer, was also tested. It is a representative of the textile composites used, among others, in the construction industry.

Therefore, representatives of composites built on the basis of fabric with polyurethane, acrylic, and Teflon coatings were examined.

The effect of the changes in the electrical parameters as a result of the generated heat was observed primarily in the structure made of Cordura, i.e., a material with a top layer made of polyurethane. In order to deepen the study of the impact of the barrier material itself on the changes in resistivity, a proprietary composite was created containing a mesh made of loosely braided bamboo fibers soaked in polyurethane resin. The observed effect was also tested on a composite containing not only polyurethane foil, but also an additional Teflon finish. In all the samples containing polyurethane, an enormous and unpredictable phenomenon of change in the resistance of the structures was observed. This indicates the limitations in the applications of this type of substrate as a base material for the production of textronic structures, which should be characterized by a strictly defined coefficient of resistance change as a function of temperature. However, due to the very good electrical properties of the thin-layer structures produced on the Cordura composite, this substrate should still be taken into account during the selection of the material on the basis of which textronic systems will be produced. The use of this type of substrate requires the introduction of a correction factor for changes in resistance as a function of the flowing current and temperature. It will be the aim of future research. 

The obtained results indicate the possibility of defining a parameter characterizing the change in layer resistance resulting from thermal changes in the substrate—the substrate resistance coefficient for layers deposited on textile substrates. The experiments carried out and obtained results show that, in the further stages of research, efforts should be made to develop a thermal coefficient for resistance changes depending on the substrate used. 

## Figures and Tables

**Table 1:** Parameters of the samples.

Sample	Name	Type	Density (g/m^2^)	Materials
Sample A	CORDURA^®^	cordura	195	nylon threads/polyurethane
Sample B	Goretex	membrane	130	nanofiber/Teflon foil
Sample C	-	non-woven fabric	130	non-woven polypropylen/acryl
Sample D	-	-	210	bamboo fibers/polyurethane
Sample E	RAINCRUCIAL^®^	cordura	155	nylon threads/polyurethane/Teflon

**Table 2:** The values of resistance of the chosen sample in the self-heating and external-heating mode.

Sample	Initial R_self-heating_ (Ω)	Max R_self-heating_ (Ω)	Final R_self-heating_ (Ω)	Initial R_external-heating_ (Ω)	Max R_external-heating_ (Ω)	Final R_external-heating_ (Ω)
Sample A	4.1	4.1	3.17	5.15	5.18	4.33
Sample D	1.08	1.12	0.98	1.1	1.1	1.0
Sample E	1.49	1.52	1.26	1.67	1.72	1.53

## Data Availability

The final data is available after contacting the authors.
